# Retrospective and Contemporary Genomic Surveillance Reveals Genotype-Specific Dengue Lineage Dynamics in Rio de Janeiro

**DOI:** 10.3390/v18101070

**Published:** 2026-09-28

**Authors:** Gabriel Schuab, Carla de Oliveira, Carolina Cardoso dos Santos, Laise de Moraes, Cintia Damasceno dos Santos, Desiree dos Santos Nunes, Juan Carlos Proença Moura, Felipe Gomes Naveca, Fernanda de Bruycker-Nogueira, Ana Maria Bispo de Filippis, Marta Giovanetti

**Affiliations:** 1 Laboratório de Arbovírus e Vírus Hemorrágicos, Instituto Oswaldo Cruz, Fundação Oswaldo Cruz, Rio de Janeiro 21040-360, Brazil; gabrielshuab@gmail.com (G.S.); oliveirasc85@gmail.com (C.d.O.); carolina.csantos2@gmail.com (C.C.d.S.); cintiadsr11@gmail.com (C.D.d.S.); desireesnunes@gmail.com (D.d.S.N.); juan.carlos.moura@gmail.com (J.C.P.M.); naveca.felipe@gmail.com (F.G.N.); februycker@gmail.com (F.d.B.-N.); bispoam@gmail.com (A.M.B.d.F.); 2 Laboratório de Medicina e Saúde Pública de Precisão, Instituto Gonçalo Moniz, Fundação Oswaldo Cruz, Salvador 40296-710, Brazil; laise.moraes@fiocruz.br; 3 Instituto Nacional de Ciência e Tecnologia em Saúde Digital (INCT DigiSaude), Instituto Gonçalo Moniz, Fundação Oswaldo Cruz, Salvador 40296-710, Brazil; 4 Department of Science and Bio-Technology, Università Campus Bio-Medico di Roma, 00128 Rome, Italy

**Keywords:** dengue virus, genomic surveillance, Rio de Janeiro

## Abstract

Dengue virus (DENV) transmission in Brazil is characterized by recurrent epidemics involving the introduction, persistence, and replacement of viral lineages. However, temporal gaps in genomic surveillance limit our understanding of how these processes shape successive epidemics in Rio de Janeiro. Here, in a retrospective observational molecular epidemiology study, we combined archived genomic data with publicly available sequences and epidemiological surveillance data to investigate the temporal dynamics of DENV serotypes and lineages in Rio de Janeiro. Archived DENV-positive samples were curated and subjected to whole-genome amplicon sequencing, and the resulting consensus sequences were analyzed together with publicly available sequences using time-scaled phylogenetic reconstruction and epidemiological data. We recovered 35 DENV genomes from samples collected in 2013, 2018, 2020, and 2021, including DENV-1 genotype V, DENV-2 genotypes II and III, and DENV-4 genotype II. Analysis of the combined datasets identified multiple Rio de Janeiro-associated clades with distinct temporal patterns. Publicly available sequences supported the broader clade structure and long-term temporal patterns independently of most genomes generated here. The newly generated genomes made genotype-specific contributions: the five DENV-1 genotype V genomes placed archived 2020–2021 samples within previously represented Rio de Janeiro-associated groups, while the single DENV-2 genotype II genome provided phylogenetic placement of a travel-associated 2018 case. In contrast, the 20 DENV-2 genotype III genomes identified a Rio de Janeiro-associated cluster not represented among the public sequences, and the nine DENV-4 genotype II genomes increased representation of the 2013 epidemic. Thus, the principal contribution of retrospective sequencing was to fill specific temporal and geographic gaps rather than to redefine the broader historical phylogenetic structure of all genotypes.

## 1. Introduction

Dengue virus (DENV) remains a major public health challenge in Brazil, where all four serotypes have circulated and recurrent epidemics have affected large populations over several decades [1]. The magnitude and timing of these epidemics are influenced by a combination of population immunity, vector abundance, human mobility, and changes in the viral populations circulating over time [2,3].

Rio de Janeiro represents an important setting in which to examine these dynamics because sustained dengue transmission, high population density, and intense connectivity with other regions of Brazil and abroad create favorable conditions for both local maintenance and the introduction of genetically distinct DENV lineages [4]. Beyond shifts in serotype predominance, dengue transmission is characterized by substantial evolutionary turnover within individual serotypes [5]. Viral lineages may persist locally across multiple transmission seasons, disappear and be replaced by genetically distinct lineages, or be repeatedly introduced from other geographic regions [6]. These processes can reshape the genetic composition of DENV populations between epidemic periods and may contribute to changes in the timing, magnitude, and geographic spread of outbreaks [7]. Distinguishing local persistence from lineage replacement and independent introductions is therefore important for understanding how dengue epidemics emerge and recur. Such distinctions, however, depend on sufficiently dense and temporally continuous genomic sampling.

Despite major advances in dengue genomic surveillance in Brazil, sequence availability remains uneven across locations and epidemic periods [4,6]. Earlier outbreaks in particular are often represented by relatively few genomic sequences, creating temporal gaps that can obscure transmission continuity and complicate the reconstruction of lineage histories [8]. Under these conditions, an apparent absence of a lineage may reflect true local extinction, replacement by another lineage, or simply insufficient sampling. Archived diagnostic collections provide an opportunity to address this limitation by recovering viral genomic information from periods that are poorly represented in public databases and integrating these historical data with contemporary surveillance.

In Rio de Janeiro, the Laboratory of Arboviruses and Hemorrhagic Viruses (LARBOH), Oswaldo Cruz Institute/Fiocruz, has conducted long-term molecular surveillance of DENV and maintains archived DENV-positive specimens collected across multiple epidemic periods [9]. Here, we conducted a retrospective observational molecular epidemiology study combining genomic data recovered from these archived samples with publicly available DENV sequences and epidemiological surveillance data. We hypothesized that sequencing archived DENV-positive samples from temporally and geographically underrepresented periods would increase genomic representation of these gaps and could recover phylogenetic diversity not represented in publicly available datasets, thereby improving the reconstruction of historical DENV lineage circulation in Rio de Janeiro. We therefore evaluated the specific contribution of retrospectively recovered genomes to the temporal, geographic, and phylogenetic reconstruction of DENV circulation in the state, distinguishing information already supported by public sequences from that added by the genomes generated in this study.

## 2. Materials and Methods

### 2.1. Data Curation, Sample Selection, and Molecular Screening

Information on dengue samples processed at LARBOH was compiled from the GAL system v2.30 (Gerenciador de Ambiente Laboratorial) and internal laboratory records. As GAL data were available only from 2015 onward, earlier records (2012–2015) were retrieved from internal spreadsheets and manually curated in Excel to standardize relevant variables. GAL datasets (2016–2021) were extracted periodically, consolidated by year, and integrated into a unified database. Filtering retained key variables, including viral serotype and quantification cycle (Cq) values, followed by standardization in R v3.5.1 [10].

The curated dataset (2012–2021) was used to characterize samples by year, serotype, Cq value, and mesoregion. Archived samples were initially selected based on historical laboratory records using a Cq threshold of ≤31, together with available sample volume, collection year, serotype, and geographic origin; up to 30 samples per year were targeted when available. A total of 94 samples meeting these criteria were retrieved from the biorepository. Access to the genetic heritage was registered in the Brazilian National System for the Management of Genetic Heritage and Associated Traditional Knowledge (SisGen) under registration number A0C0D2F. RNA was re-extracted using the MagMAX™ Pathogen RNA/DNA Kit (Thermo Fisher Scientific, Waltham, MA, USA) and Extracta Kit MPTA-PV48-B (Loccus, Cotia, São Paulo, Brazil) on the KingFisher™ Flex system (Thermo Fisher Scientific Oy, Vantaa, Finland), following the manufacturers’ protocols. DENV detection and confirmation were performed by real-time RT-PCR using an in-house protocol adapted from Santiago et al. [11]. Reactions (25 μL) included serotype-specific primers and probes, and cycling conditions consisted of reverse transcription (45 °C, 30 min), enzyme activation (95 °C, 2 min), and 45 amplification cycles. Cq values were determined using QuantStudio™ software v1.6. Following re-extraction and RT-qPCR confirmation, 55 of the 94 retrieved samples met the laboratory inclusion criterion (Cq ≤ 31) and proceeded to sequencing. A final database including geographic location, mesoregion, Cq value, collection year, and serotype was generated, and spatial analyses were performed in RStudio v4.4.2 using geobr and ggplot2 [12,13].

### 2.2. cDNA Synthesis, Genomic Amplification, and Sequencing

Extracted RNA was reverse transcribed using the ProtoScript^®^ II First Strand cDNA Synthesis Kit (New England Biolabs, Ipswich, MA, USA). cDNA was amplified by multiplex PCR using serotype-specific primer schemes covering the full DENV genome (DENV-1 to DENV-4). For DENV-2, an additional primer set targeting the Cosmopolitan genotype was included. Amplifications were performed in two pools (A and B) per sample. PCR products were purified (AMPure XP (Beckman Coulter, Brea, CA, USA)), quantified (Qubit 4, dsDNA HS kit (Beckman Coulter, Brea, CA, USA)), and normalized to 50 ng. For nanopore sequencing, samples were barcoded (BC01–BC96), pooled, purified, and prepared using the Rapid Barcoding 96 (V14) kit (Oxford Nanopore Technologies, Oxford, UK). Libraries were sequenced on an R10.4.1 flow cell using the MinION platform (MinKNOW) (Oxford Nanopore Technologies, Oxford, UK) [14]. For Illumina sequencing, libraries were prepared using the Illumina COVIDSeq kit (Illumina, San Diego, CA, USA), replacing the original SARS-CoV-2 primer pools with DENV serotype-specific primers, as previously described for DENV whole-genome sequencing [15,16]. Libraries were sequenced on a NextSeq instrument (Illumina, San Diego, CA, USA) using a P1 cartridge, and FASTQ files were generated using BaseSpace v7.50.0. The serotype-specific amplicon schemes were designed to span the DENV genome rather than target a single genomic fragment. Genome recovery varied among samples, and some consensus sequences therefore contained regions of missing sequence data.

### 2.3. Bioinformatic Processing, Genome Assembly, and Phylogenetic Analysis

Nanopore POD5 files were basecalled with Dorado v1.3.0 [14] using the super-accurate model. Reads were quality-filtered (Q ≥ 10), demultiplexed, trimmed, and length-filtered (BBDuk v39.94) [17]. Genome assembly was performed with minimap2 v2.26 [18] using GenBank references [19]. Primer trimming (iVar v1.4.4) [20], polishing, and variant calling (Clair3 v2.0.1) [21] were applied. Positions with coverage <10 were masked, and consensus genomes were generated with BCFtools v1.23.1 [22]. Assembly metrics were obtained using SAMtools/HTSlib v1.19.2 and Seqtk v1.5 [23].

Illumina reads were processed with fastp v1.1.0 [24] for quality filtering. Filtered reads were mapped to selected reference genomes using BWA v2.2.3. For both sequencing platforms, positions with read depth <10× were masked as N and retained as missing data in alignments spanning the DENV genome coordinate space. Alignment columns were not removed solely because they were uncovered in individual samples. Sequences with <60% genome coverage were excluded from downstream phylogenetic analyses, whereas sequences with ≥60% coverage were retained as an inclusion criterion for subsequent quality-control and temporal-signal assessment. This threshold was used pragmatically to retain informative archived sequences while excluding highly incomplete genomes and was not used to classify sequences as complete genomes. Assembly and coverage statistics were calculated using SAMtools/HTSlib and Seqtk. Phylogenetic analyses were performed separately for DENV-1 genotype V, DENV-2 genotype II, DENV-2 genotype III, and DENV-4 genotype II. The newly generated sequences—DENV-2 genotype III (*n* = 20), DENV-4 genotype II (*n* = 9), DENV-1 genotype V (*n* = 5), and DENV-2 genotype II (*n* = 1)—were combined with publicly available DENV sequences retrieved from GenBank and GISAID for each corresponding genotype. Public sequences were retained if they could be assigned to the genotype under analysis, represented genome-scale sequence records, and had complete collection-date and geographic-origin metadata required for temporal analysis and geographic annotation. The reference datasets comprised 809 DENV-1 genotype V sequences, 2476 DENV-2 genotype II sequences, 761 DENV-2 genotype III sequences, and 1338 DENV-4 genotype II sequences. Sequence datasets were aligned using MAFFT v7.505 [25], and maximum-likelihood trees were inferred with IQ-TREE2 v2.0.7 under the GTR+G4+F substitution model. Rio de Janeiro-associated clades were identified from the maximum-likelihood topology as monophyletic groupings containing Rio de Janeiro sequences and supported by SH-aLRT values. Temporal molecular-clock signal was assessed using TempEst v1.5.3 [26] and the clock functionality implemented in TreeTime v0.11.1 [27]. Maximum-likelihood trees were subsequently transformed into time-scaled phylogenies using TreeTime. Sequences showing substantial deviation from the molecular-clock expectation were iteratively excluded during temporal quality control using the ape package in R v4.4.2. Nine newly generated sequences were removed during this step. Final trees were annotated with geographic metadata and visualized in R using ggtree v3.14.0, treeio 1.30.0, and associated packages.

### 2.4. Epidemiological Analysis

Epidemiological analyses were conducted using secondary data on reported dengue cases in the state of Rio de Janeiro from 2014 to 2025. Data were obtained from the Notifiable Diseases Information System (SINAN) and included records aggregated by year of notification, municipality of residence, and viral serotype. Surveillance data were extracted in October 2025. Because the 2025 dataset was incomplete at the time of extraction, data for 2025 were considered provisional. Datasets were standardized through variable harmonization, data restructuring, and handling of missing values. Temporal trends were assessed using the annual distribution of reported cases, while spatial distribution was assessed by calculating the cumulative number of cases per municipality over the study period. Viral serotype circulation was analyzed by year of notification, restricted to records with available serotype information. The proportion of notifications with available serotype information was calculated to assess the extent of virological characterization within routine surveillance. All analyses and data visualizations were performed in the R environment.

## 3. Results

### 3.1. Epidemiological Context and Gaps in Virological Surveillance in Rio de Janeiro (2014–2025)

Dengue notifications in Rio de Janeiro State showed marked interannual variation between 2014 and 2025, with epidemic peaks in 2015, 2016, 2019, 2023, and particularly 2024, which represented the largest epidemic during the analyzed period (Figure 1A). Notifications declined during 2020–2021 and increased again from 2022 onward. These fluctuations provide the temporal context for the periods represented by the archived specimens and illustrate the heterogeneous intensity of dengue transmission across successive epidemic years.

Dengue notifications were reported across nearly all municipalities of Rio de Janeiro State, although cumulative case burden was spatially heterogeneous (Figure 1B). This geographic heterogeneity provides context for assessing the spatial representation of the archived samples and the genomes recovered in this study.

In contrast to the large number of reported dengue cases, virological characterization was limited. Only approximately 5% of notifications between 2014 and 2025 had associated serotype information in SINAN, and 52% of all records with available serotype information were concentrated in 2024 (Figure 1C). Thus, the epidemiological surveillance data reveal a substantial gap between case notification and virological characterization, limiting the ability to reconstruct changes in serotype and lineage circulation across successive epidemic periods. This gap provides the epidemiological rationale for recovering and sequencing archived DENV-positive specimens from underrepresented years and locations.

### 3.2. Retrospective Sample Curation and Laboratory Triage

Archived dengue-positive samples collected between 2013 and 2021 and stored in the laboratory biobank were retrospectively curated to expand temporal genomic coverage. Following RNA re-extraction and RT-qPCR confirmation, 55 of the 94 retrieved samples met the laboratory inclusion criteria and proceeded to sequencing (Table 1). These 55 samples spanned four collection years and fourteen municipalities across Rio de Janeiro State (Table 1 and Table S1). Three DENV serotypes were represented. DENV-4 predominated among samples collected in 2013, whereas DENV-2 accounted for most samples from 2021, consistent with the epidemiological patterns observed during these periods (Table 1). The largest numbers of samples originated from Volta Redonda (*n* = 21) and Rio de Janeiro (*n* = 15), both of which reported substantial dengue notification burdens during the corresponding years (Table 1). Overall, Cq values ranged from 17.18 to 30.84, with median values varying by serotype and year of collection, reflecting differences in viral load among archived specimens (Table 1). Most curated samples were derived from urban municipalities with historically high dengue incidence, consistent with more intensive diagnostic activity in densely populated settings. Demographic data indicated that sampled individuals ranged in age from 4 to 82 years, encompassing pediatric, adult, and elderly groups. Most samples were collected from adolescents and adults, particularly individuals aged 16–55 years (Table S1). Both sexes were represented, with a slight predominance of male patients. Overall, the demographic profile of the curated samples was consistent with the age and sex distribution typically reported for dengue cases in Rio de Janeiro State.

### 3.3. Genome Recovery and Expansion of Temporal, Geographic, and Genomic Surveillance Coverage

Sequencing generated DENV consensus sequences with heterogeneous levels of genome coverage across retrospective samples. Of the 55 samples that proceeded to sequencing, 44 produced consensus sequences with ≥60% genome coverage and were therefore eligible for downstream phylogenetic analysis. Following phylogenetic and temporal quality control, nine sequences were excluded as molecular-clock outliers, resulting in 35 consensus sequences retained for the final time-scaled phylogenetic analyses, corresponding to a final yield of 37% (35/94). The recovery of these retrospective genomes expanded the temporal, geographic, and genomic coverage of DENV in the state of Rio de Janeiro (Figure 2A). These 35 genomes were distributed across 2013, 2018, 2020, and 2021, with the highest number in 2021 (*n* = 16), followed by 2013 and 2020 (*n* = 9 each), whereas 2018 contributed a single genome (*n* = 1) (Figure 2B). This distribution reflects both sample availability and the intensity of viral circulation during these periods, while improving the historical representativeness of the dataset. Spatial expansion was also evident, with sequences recovered from multiple municipalities and mesoregions across the state (Figure 2). The Metropolitana mesoregion accounted for the largest number of sequences, particularly in 2013 (*n* = 6) and 2021 (*n* = 9). The Sul Fluminense mesoregion contributed substantially in 2020 (*n* = 9) and 2021 (*n* = 6), while additional sequences were identified in Baixadas Litorâneas (one in 2013 and one in 2021) and Norte Fluminense (two in 2013).

From a genomic perspective, four distinct groups were identified: DENV-2 genotype III (*n* = 20), DENV-4 genotype II (*n* = 9), DENV-1 genotype V (*n* = 5), and DENV-2 genotype II (*n* = 1) (Figure 2C). The contribution of these retrospective genomes was uneven across genotypes. Their principal value was to increase representation of specific temporal and geographic gaps, with the strongest additional phylogenetic information obtained for DENV-2 genotype III, whereas the DENV-1 genotype V and DENV-2 genotype II genomes primarily provided additional sampling within phylogenetic patterns already represented by public sequences.

### 3.4. Lineage-Specific Phylogenetic and Temporal Dynamics of DENV in Rio de Janeiro

The time-scaled phylogenetic analysis of different DENV serotypes and genotypes revealed distinct patterns consistent with viral introduction, lineage persistence, and temporal turnover in Rio de Janeiro. Clades were delimited based on Rio de Janeiro-associated lineages, allowing a more focused interpretation of state-level viral dynamics and of the contribution of the genomes generated in this study. Figure 3 summarizes the temporal phylogenies of DENV-1 genotype V, DENV-2 genotype II/Cosmopolitan, DENV-2 genotype III, and DENV-4 genotype II, shown in panels A–D, respectively.

For DENV-1 genotype V, seven Rio de Janeiro-associated clades were identified, spanning a broad temporal range from the late 1980s to 2024. Clade RJ ii, dated between 1988 and 2001, represents a historical lineage associated with the state, whereas clades RJ i, RJ v, and RJ vi indicate subsequent episodes of circulation, mainly covering the periods 2010–2016, 2010–2012, and 2011–2024, respectively. The presence of more recent clades, including RJ iii and RJ iv, both dated between 2021 and 2024, as well as RJ vii, dated between 2023 and 2024, suggests the continued circulation of DENV-1 lineages in Rio de Janeiro during recent epidemic periods. The broader public dataset supports the presence of multiple Rio de Janeiro-associated groupings over time. The five genomes generated in this study do not independently demonstrate multiple introductions; rather, they place archived 2020–2021 samples within previously represented Rio de Janeiro-associated groups.

For DENV-2 genotype II/Cosmopolitan, five Rio de Janeiro-associated clades were observed. Clade RJ i, dated to 2018, contains the single genome generated in this study and supports only the phylogenetic placement of this travel-associated case; it does not support conclusions regarding lineage persistence or additional introductions. Clades RJ ii to RJ v, dated 2023–2024, are supported by publicly available sequences and provide the broader context of recent Cosmopolitan-genotype circulation in Brazil. The single genome generated in this study supports the phylogenetic placement of the travel-associated 2018 detection but does not independently support inference of lineage persistence, sustained local transmission, or additional introductions. The 2023–2024 Rio de Janeiro-associated clades are supported by publicly available sequences and provide the broader context of more recent DENV-2 genotype II/Cosmopolitan circulation. Thus, the contribution of the newly generated sequence is restricted to documenting the phylogenetic context of the 2018 travel-associated case.

For DENV-2 genotype III, the analysis revealed five Rio de Janeiro-associated clades distributed between 1990 and 2024. Clade RJ i, dated between 1990 and 2009, represents the oldest lineage associated with the state, reflecting the historical circulation of this genotype in the Americas and Brazil. Clade RJ ii, dated between 2007 and 2011, indicates a second period of circulation following the older grouping. Clades RJ iv, RJ v, and RJ iii represent more recent events, dated to 2019, 2020–2021, and 2020–2024, respectively. The 20 DENV-2 genotype III genomes generated in this study substantially increased representation of 2020–2021 circulation. Notably, clade RJ v was composed exclusively of genomes generated in this study and was therefore not represented in the public dataset used here. This cluster constitutes the clearest phylogenetic structure uniquely recovered through retrospective sequencing in the present study. The broader temporal pattern of DENV-2 genotype III, however, including multiple Rio de Janeiro-associated clades across different periods, remains supported by the combined historical dataset and should not be attributed solely to the newly generated genomes.

For DENV-4 genotype II, four Rio de Janeiro-associated clades were identified, all concentrated between 2011 and 2013. Clade RJ i, dated between 2011 and 2013, presents the first sequence associated with the state, whereas clades RJ ii, RJ iii, and RJ iv, dated to 2012–2013, 2012, and 2013, respectively, indicate temporal substructure within the same epidemic period. In contrast to the other genotypes, DENV-4 genotype II did not show recent Rio de Janeiro-associated clades in the analyzed tree, suggesting that its circulation in the state was concentrated during the period of introduction and early establishment in Brazil. The nine retrospective DENV-4 genotype II genomes increased the temporal and geographic representation of the 2013 epidemic. However, the broader historical pattern of DENV-4 genotype II circulation during 2011–2013 was already represented by publicly available sequences; therefore, the newly generated genomes improve sampling of this period rather than independently demonstrating introduction or persistence.

Considered without the genomes generated in this study, the publicly available datasets would still support the broader historical pattern of multiple Rio de Janeiro-associated DENV-1 genotype V and DENV-2 genotype III clades, recent DENV-2 genotype II/Cosmopolitan circulation, and the concentration of DENV-4 genotype II-associated clades around 2011–2013. The retrospective genomes added different levels of information across genotypes. The five DENV-1 genotype V genomes placed archived 2020–2021 samples within previously represented groups, and the single DENV-2 genotype II genome provided phylogenetic placement of the 2018 travel-associated detection. In contrast, the 20 DENV-2 genotype III genomes revealed a Rio de Janeiro-associated cluster not represented among the public sequences, while the nine DENV-4 genotype II genomes increased temporal and geographic representation of the 2013 epidemic. Thus, retrospective sequencing primarily filled specific sampling gaps, with its strongest additional phylogenetic contribution observed for DENV-2 genotype III.

## 4. Discussion

Recurrent dengue epidemics in endemic settings arise from the interaction of epidemiological, ecological, and evolutionary processes that continuously reshape the composition of circulating DENV populations [1,2,3]. Beyond changes in serotype predominance, viral populations may undergo lineage persistence, co-circulation, replacement, and introduction of genetically distinct viruses, as documented for different DENV serotypes in Brazil and elsewhere [28,29,30]. Distinguishing among these processes is important for understanding how dengue transmission is maintained and renewed over time, but requires sufficiently continuous genomic sampling to differentiate true lineage turnover from apparent changes caused by gaps in surveillance [4,6].

The phylogenetic patterns reconstructed for Rio de Janeiro indicate markedly different evolutionary histories among the DENV genotypes analyzed. DENV-1 genotype V and DENV-2 genotype III included multiple Rio de Janeiro-associated clades distributed across successive epidemic periods, with patterns compatible with lineage persistence and turnover. DENV-2 genotype II/Cosmopolitan showed temporally distinct detections, whereas DENV-4 genotype II displayed a substantially more restricted temporal pattern concentrated around 2011–2013.

The temporal structure observed for DENV-1 genotype V is consistent with previous evidence of the co-circulation and replacement of distinct DENV-1 lineages in Brazil, including Rio de Janeiro [28,29]. Similarly, the temporal distribution of DENV-2 genotype III clades is consistent with previous studies documenting re-introduction, persistence, and lineage replacement of DENV-2 in Brazil [30]. DENV-2 genotype II/Cosmopolitan showed a contrasting pattern. The 2018 Rio de Janeiro sequence was phylogenetically separated from the more recent Rio de Janeiro-associated groupings and was linked epidemiologically to international travel, supporting its interpretation as an isolated detection rather than evidence of sustained local transmission [31]. DENV-4 genotype II presented a different evolutionary history, with Rio de Janeiro-associated clades concentrated around 2011–2013. This period broadly corresponds to the re-emergence and subsequent dissemination of DENV-4 in Brazil [32,33]. Previous phylogeographic analyses have demonstrated multiple introductions and regional dissemination of DENV-4 genotype II during the 2012–2013 epidemic period [32]. Although no comparable recent Rio de Janeiro-associated DENV-4 genotype II clades were observed in our dataset, their absence cannot by itself demonstrate local extinction, particularly given heterogeneous genomic sampling over time. The contrasting patterns among DENV-1, DENV-2, and DENV-4 nevertheless reinforce the importance of examining lineage-level dynamics rather than relying exclusively on changes in serotype predominance.

The public datasets provide the broader clade structure and long-term temporal context, whereas the genomes generated in this study contributed primarily by filling specific sampling gaps. The five DENV-1 genotype V genomes placed archived 2020–2021 samples within previously represented Rio de Janeiro-associated groups, and the single DENV-2 genotype II genome placed the 2018 travel-associated case without supporting inference of lineage persistence. In contrast, the 20 DENV-2 genotype III genomes added representation of 2020–2021 circulation and identified a Rio de Janeiro-associated cluster absent from the public sequences, while the nine DENV-4 genotype II genomes increased temporal and geographic representation of the 2013 epidemic. These genotype-specific contributions show why retrospective genomic recovery complements, rather than replaces, prospective surveillance and public datasets [4,6,31].

The epidemiological findings further reinforce the need for greater continuity in virological surveillance. Dengue notifications in Rio de Janeiro varied substantially between 2014 and 2025, including a major epidemic in 2024, within the broader context of recurrent and spatially connected dengue transmission in Brazil [1,2]. However, only approximately 5% of notified cases in the analyzed surveillance dataset had associated serotype information. Limited virological characterization restricts the ability to determine whether changes in epidemic magnitude coincide with shifts in serotype or lineage composition and reduces the pool of representative specimens available for genomic analysis. Increasing serotyping and genomic sequencing across municipalities and during both epidemic and interepidemic periods would provide a stronger basis for identifying changes in circulating viral populations and interpreting their relationship with epidemic dynamics.

Accordingly, the evidentiary contribution of the newly generated genomes should not be considered equivalent across genotypes: inference for DENV-1 genotype V and DENV-2 genotype II remains driven primarily by the broader public dataset, whereas the clearest phylogenetic information uniquely added by this study derives from DENV-2 genotype III. Of the 94 archived samples retrieved, 39 did not meet the RT-qPCR inclusion criterion after RNA re-extraction, 11 of the 55 samples that proceeded to sequencing did not meet the ≥60% genome-coverage criterion, and nine of the 44 eligible sequences were excluded during temporal quality control. The final yield of 35/94 (37%) therefore reflects both the quality and integrity of the archived starting material and the application of downstream genomic and temporal quality-control criteria; it should not be interpreted as a measure of sequencing efficiency alone or as a universal expected yield for retrospective genomic surveillance.

These findings support four operational surveillance actions for Rio de Janeiro. First, genomic surveillance should include RT-qPCR-positive samples from multiple mesoregions during both epidemic and interepidemic periods, rather than concentrating sequencing in periods or locations with the highest diagnostic activity. Second, surveillance programs should periodically assess genomic representation by year, municipality or mesoregion, and serotype, and prioritize archived samples from strata with few or no available genomes. Third, genomic records should be routinely linked to collection date, municipality, serotype, Cq value, and travel history, allowing isolated travel-associated detections to be distinguished from patterns compatible with sustained local transmission. Fourth, retrospective sequencing should be used to investigate apparent temporal gaps before interpreting the absence of a lineage as evidence of local disappearance or replacement. Together, these actions directly address the temporal and geographic sampling gaps, limited serotyping coverage, and the isolated travel-associated DENV-2 genotype II detection identified in this study.

## 5. Conclusions

DENV circulation in Rio de Janeiro showed genotype-specific phylogenetic patterns compatible with lineage persistence, turnover, and the emergence of genetically distinct viral populations across successive epidemic periods. Publicly available sequences supported the broader clade structure and long-term temporal patterns, whereas the archived genomes generated in this study made genotype-specific contributions rather than uniformly altering the historical reconstruction. Their clearest additional phylogenetic contribution was the identification of a DENV-2 genotype III Rio de Janeiro-associated cluster not represented among public sequences, while the DENV-1 genotype V, DENV-2 genotype II, and DENV-4 genotype II genomes primarily increased representation of specific archived periods or detections. Based on these findings, genomic surveillance in Rio de Janeiro should maintain sequencing across epidemic and interepidemic periods and across geographically distinct mesoregions, periodically identify years, locations, and serotypes with limited genomic representation and use archived material to address these gaps, and routinely link sequence data with collection date, municipality, serotype, Cq value, and travel history. This approach would help distinguish isolated introductions from sustained local transmission and reduce the risk of interpreting gaps in genomic sampling as lineage disappearance or replacement.

## Figures and Tables

**Figure 1:**
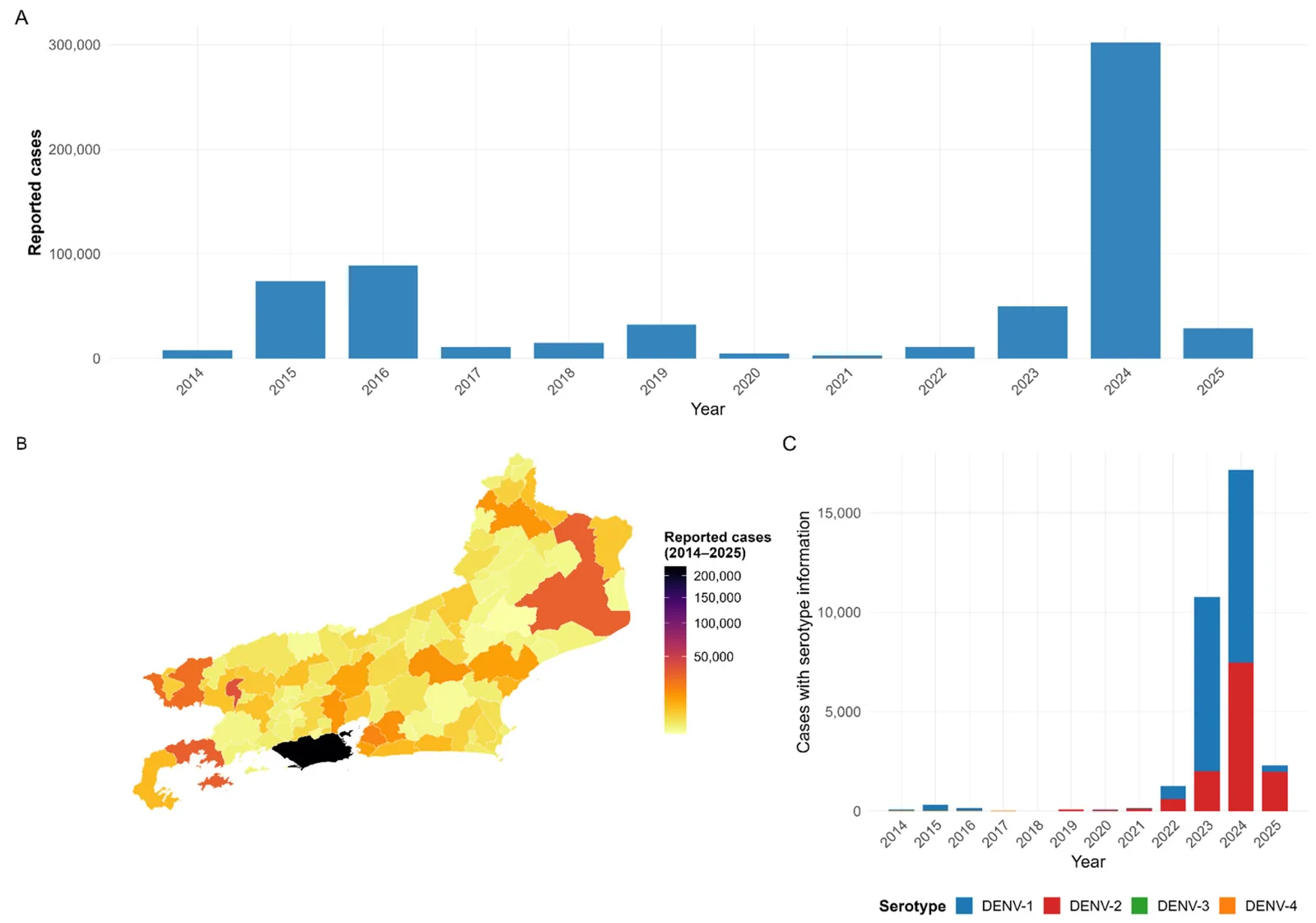
Epidemiological context and virological surveillance gaps for dengue in Rio de Janeiro State, Brazil, 2014–2025. (**A**) Annual number of reported dengue cases; (**B**) spatial distribution of cumulative dengue notifications by municipality; and (**C**) annual distribution of DENV serotypes among notified cases with available serotype information. The figure highlights the marked temporal and spatial heterogeneity of dengue transmission and the limited availability of serotype information in routine surveillance data.

**Figure 2:**
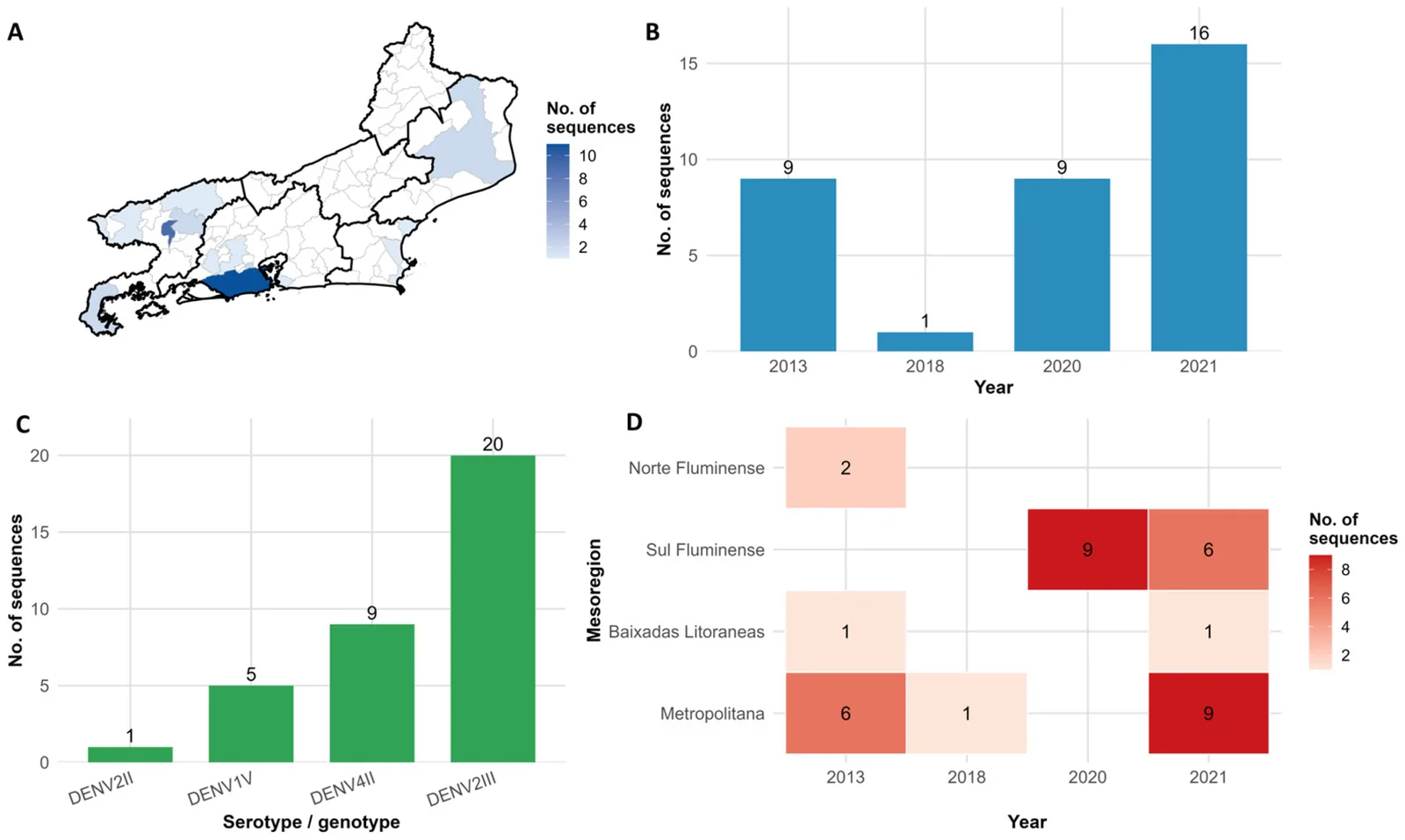
Temporal, geographic, and genomic coverage of DENV sequences recovered in the state of Rio de Janeiro. (**A**) municipal distribution of the sequences; (**B**) temporal distribution by year; (**C**) composition by serotype/genotype; and (**D**) distribution of the sequences by mesoregion over the analyzed period.

**Figure 3:**
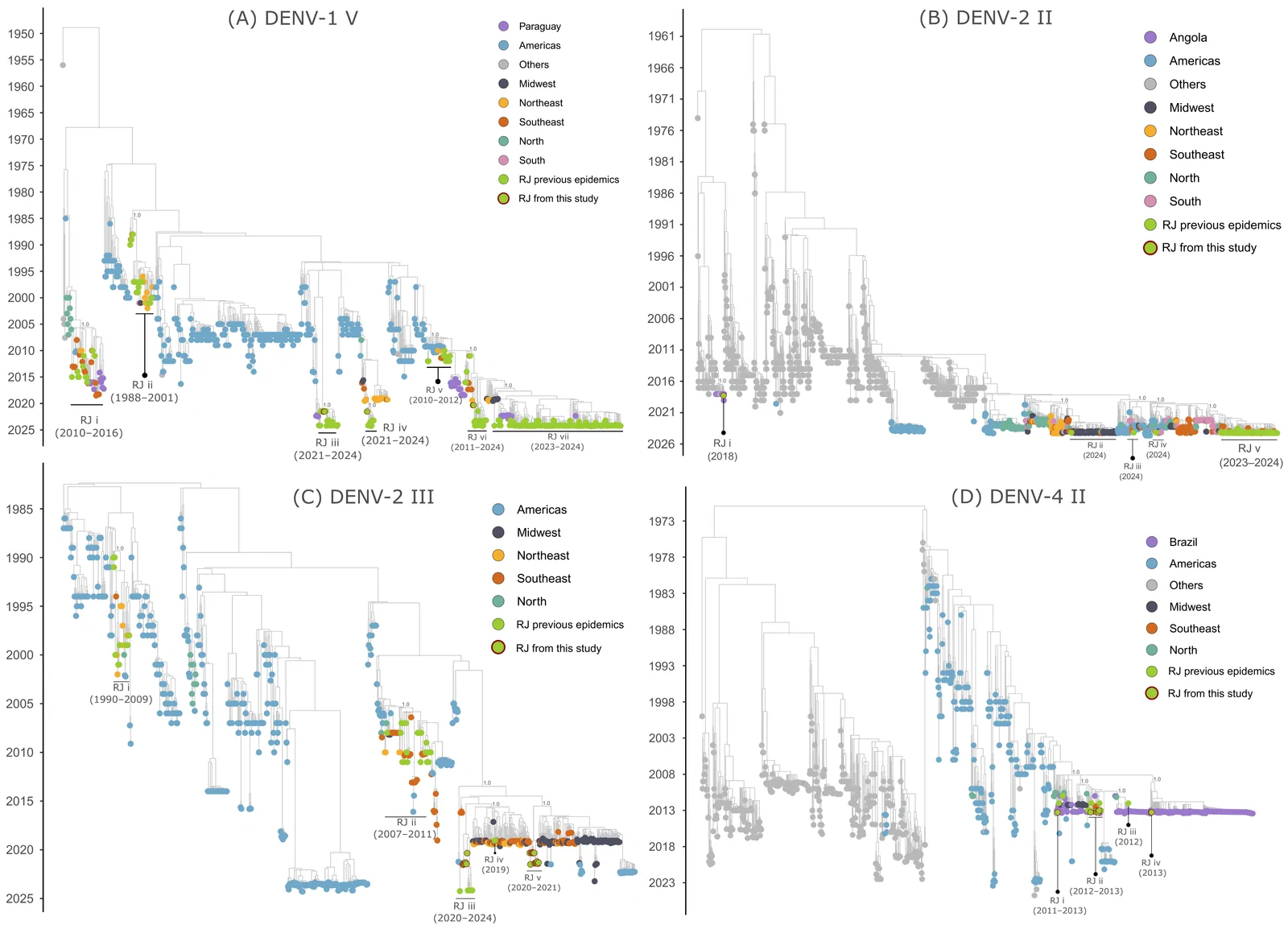
Time-scaled phylogenetic reconstruction of DENV lineages associated with Rio de Janeiro. Panels show the temporal phylogenies of DENV-1 genotype V ((**A**); *n* = 805 tips), DENV-2 genotype II ((**B**); *n* = 2380 tips), DENV-2 genotype III ((**C**); *n* = 775 tips), and DENV-4 genotype II ((**D**); *n* = 1285 tips). Tips are colored according to geographic origin, with Rio de Janeiro sequences generated in this study highlighted as RJ_this_study. Rio de Janeiro-associated clades are labeled in each tree. SH-aLRT support values are shown at key internal nodes, and all highlighted clades received maximum support (SH-aLRT = 100%). The *y*-axis represents time in years.

**Table 1:** Retrospectively curated dengue-positive samples that met laboratory inclusion criteria and proceeded to sequencing (*n* = 55). The table summarizes the distribution of selected samples (*n*) by serotype, collection year, municipality, and mesoregion, together with median Cq values and corresponding minimum–maximum ranges.

Serotype	Year	Municipality	Mesoregion	*n*	Cq (Median [Min–Max])
DENV-1	2020	Parati	Sul Fluminense	2	25.00 (19.16–30.84)
DENV-1	2021	Nova Iguaçu	Metropolitana	1	28.28
DENV-1	2021	Rio de Janeiro	Metropolitana	2	23.92 (17.18–30.66)
DENV-1	2021	São João de Meriti	Metropolitana	1	27.24
DENV-2	2018	Rio de Janeiro	Metropolitana	1	20.90
DENV-2	2020	Valença	Sul Fluminense	2	26.71 (24.49–28.93)
DENV-2	2020	Volta Redonda	Sul Fluminense	17	25.78 (21.28–30.84)
DENV-2	2021	Barra do Piraí	Sul Fluminense	2	24.31 (22.87–25.76)
DENV-2	2021	Barra Mansa	Sul Fluminense	2	28.45 (28.42–28.48)
DENV-2	2021	Cabo Frio	Baixadas Litorâneas	1	24.82
DENV-2	2021	Parati	Sul Fluminense	1	28.14
DENV-2	2021	Resende	Sul Fluminense	1	25.73
DENV-2	2021	Rio de Janeiro	Metropolitana	9	23.65 (20.71–26.23)
DENV-2	2021	Volta Redonda	Sul Fluminense	4	24.28 (21.40–29.61)
DENV-4	2013	Campos dos Goytacazes	Norte Fluminense	2	27.75 (24.93–30.52)
DENV-4	2013	Niterói	Metropolitana	1	29.2
DENV-4	2013	Rio das Ostras	Baixadas Litorâneas	1	26.66
DENV-4	2013	Rio de Janeiro	Metropolitana	3	26.08 (22.96–27.99)
DENV-4	2013	São Joao de Meriti	Metropolitana	1	30.36
DENV-4	2013	Seropédica	Metropolitana	1	29.54

## Data Availability

The sequences generated in this study were deposited in GISAID EpiArbo and are available under accession numbers EPI_ISL_20485426 to EPI_ISL_20485459 and EPI_ISL_20513594. Other results are original contributions and are included in the article. Further inquiries can be directed to the corresponding author.

## Supplementary Materials

The following supporting information can be downloaded at: https://www.mdpi.com/article/10.3390/v18101070/s1, Table S1. Epidemiological and sequencing details of DENV samples from Rio de Janeiro, Brazil.

## References

[1] Fares R.C.G., Souza K.P.R., Añez G., Rios M. (2015). Epidemiological Scenario of Dengue in Brazil. BioMed Res. Int..

[2] Churakov M., Villabona-Arenas C.J., Kraemer M.U.G., Salje H., Cauchemez S. (2019). Spatio-temporal dynamics of dengue in Brazil: Seasonal travelling waves and determinants of regional synchrony. PLoS Negl. Trop. Dis..

[3] Barmak D.H., Dorso C.O., Otero M., Solari H.G. (2011). Dengue epidemics and human mobility. Phys. Rev. E.

[4] Schuab G., Tosta S., Moreno K., Fonseca V., Santos L.A., Slavov S.N., Kashima S., Ciccozzi M., Lourenço J., Cella E. (2024). Exploring the urban arbovirus landscape in Rio de Janeiro, Brazil: Transmission dynamics and patterns of disease spread. Lancet Reg. Health—Am..

[5] Zhang C., Mammen M.P., Chinnawirotpisan P., Klungthong C., Rodpradit P., Monkongdee P., Nimmannitya S., Kalayanarooj S., Holmes E.C. (2005). Clade replacements in dengue virus serotypes 1 and 3 are associated with changing serotype prevalence. J. Virol..

[6] Adelino T.É.R., Giovanetti M., Fonseca V., Xavier J., de Abreu Á.S., Nascimento V.A.D., Demarchi L.H.F., Oliveira M.A.A., da Silva V.L., de Mello A.L.E.S. (2021). Field and classroom initiatives for portable sequence-based monitoring of dengue virus in Brazil. Nat. Commun..

[7] Barcelos Figueiredo L., Sakamoto T., Leomil Coelho L.F., de Oliveira Rocha E.S., Gomes Cota M.M., Ferreira G.P., de Oliveira J.G., Kroon E.G. (2014). Dengue virus 2 American-Asian genotype identified during the 2006/2007 outbreak in Piauí, Brazil reveals a Caribbean route of introduction and dissemination of dengue virus in Brazil. PLoS ONE.

[8] de Souza U.J.B., Macedo Y.S.M., Santos R.N., Cardoso F.D.P., Galvão J.D., Gabev E.E., Franco A.C., Roehe P.M., Spilki F.R., Campos F.S. (2023). Circulation of Dengue Virus Serotype 1 Genotype V and Dengue Virus Serotype 2 Genotype III in Tocantins State, Northern Brazil, 2021–2022. Viruses.

[9] Laboratory of Arboviruses and Hemorrhagic Viruses | Oswaldo Cruz Institute. https://www.ioc.fiocruz.br/en/larboh.

[10] Hackenberger B.K. (2020). R software: Unfriendly but probably the best. Croat. Med. J..

[11] Santiago G.A., González G.L., Cruz-López F., Muñoz-Jordan J.L. (2019). Development of a Standardized Sanger-Based Method for Partial Sequencing and Genotyping of Dengue Viruses. J. Clin. Microbiol..

[12] Wickham H. (2016). ggplot2.

[13] Pereira R.H.M., Gonçalves C.N., Araujo P.H.F., Carvalho G.D., Arruda R.A., Nascimento I., Costa B.S.P., Cavedo W.S., Andrade P.R., Silva A. (2025). R Package.

[14] Oxford Nanopore Technologies (2026). Dorado. https://nanoporetech.com/software/other/dorado.

[15] Abera A., Tegally H., Tasew G., Wilkinson E., Ali A., Regasa F., Bitew M., Belachew M., Chabuka L., Mwanyika G. (2025). Genomic Characterization of Circulating Dengue Virus, Ethiopia, 2022–2023. Emerg. Infect. Dis..

[16] Sacchetto L., Marques B.C., Banho C.A., Bernardi V., Estofolete C.F., Dos Santos C.L.S., Timenetsky M.D.C.S.T., de Lacerda M.V.G., Freitas A.C., Pereira D.B. (2025). Dengue virus genetic diversity in unvaccinated and vaccinated dengue-infected individuals: An observational analysis of the Butantan-DV phase 3 trial in Brazil. Lancet Reg. Health Am..

[17] BBDuk Guide. Archive. https://archive.jgi.doe.gov/data-and-tools/software-tools/bbtools/bb-tools-user-guide/bbduk-guide/.

[18] Li H. (2018). Minimap2: Pairwise alignment for nucleotide sequences. Bioinformatics.

[19] Sayers E.W., Cavanaugh M., Clark K., Pruitt K.D., Sherry S.T., Yankie L., Karsch-Mizrachi I. (2025). GenBank 2025 update. Nucleic Acids Res..

[20] Castellano S., Cestari F., Faglioni G., Tenedini E., Marino M., Artuso L., Manfredini R., Luppi M., Trenti T., Tagliafico E. (2021). iVar, an Interpretation-Oriented Tool to Manage the Update and Revision of Variant Annotation and Classification. Genes.

[21] Su J., Zheng Z., Ahmed S.S., Lam T.W., Luo R. (2022). Clair3-trio: High-performance Nanopore long-read variant calling in family trios with trio-to-trio deep neural networks. Brief. Bioinform..

[22] Danecek P., Bonfield J.K., Liddle J., Marshall J., Ohan V., Pollard M.O., Whitwham A., Keane T., McCarthy S.A., Davies R.M. (2021). Twelve years of SAMtools and BCFtools. Gigascience.

[23] Shen W., Le S., Li Y., Hu F. (2016). SeqKit: A Cross-Platform and Ultrafast Toolkit for FASTA/Q File Manipulation. PLoS ONE.

[24] Gu J. (2018). fastp: An ultra-fast all-in-one FASTQ preprocessor. Bioinformatics.

[25] Katoh K. (2002). MAFFT: A novel method for rapid multiple sequence alignment based on fast Fourier transform. Nucleic Acids Res..

[26] Rambaut A., Lam T.T., Max Carvalho L., Pybus O.G. (2016). Exploring the temporal structure of heterochronous sequences using TempEst (formerly Path-O-Gen). Virus Evol..

[27] Sagulenko P., Puller V., Neher R.A. (2018). TreeTime: Maximum-likelihood phylodynamic analysis. Virus Evol..

[28] de Bruycker-Nogueira F., Souza T.M.A., Chouin-Carneiro T., da Costa Faria N.R., Santos J.B., Torres M.C., Ramalho I.L.C., de Aguiar S.F., Nogueira R.M.R., de Filippis A.M.B. (2018). DENV-1 Genotype V in Brazil: Spatiotemporal dispersion pattern reveals continuous co-circulation of distinct lineages until 2016. Sci. Rep..

[29] dos Santos F.B., Nogueira F.B., Castro M.G., Nunes P.C., de Filippis A.M., Faria N.R., Simões J.B., Sampaio S.A., Santos C.R., Nogueira R.M. (2011). First report of multiple lineages of dengue viruses type 1 in Rio de Janeiro, Brazil. Virol. J..

[30] Torres M.C., de Bruycker Nogueira F., Fernandes C.A., Louzada Silva Meira G., Ferreira de Aguiar S., Chieppe A.O., Bispo de Filippis A.M. (2019). Re-introduction of dengue virus serotype 2 in the state of Rio de Janeiro after almost a decade of epidemiological silence. PLoS ONE.

[31] Gräf T., Ferreira C.D.N., de Lima G.B., de Lima R.E., Machado L.C., Campos T.L., Schemberger M.O., Faoro H., Paiva M.H.S., Bezerra M.F. (2023). Multiple introductions and country-wide spread of DENV-2 genotype II (Cosmopolitan) in Brazil. Virus Evol..

[32] Ortiz-Baez A.S., Cunha M.D.P., Vedovello D., Colombo T.E., Nogueira M.L., Villabona-Arenas C.J., Zanotto P.M.A. (2019). Origin, tempo, and mode of the spread of DENV-4 Genotype IIB across the state of São Paulo, Brazil during the 2012–2013 outbreak. Mem. Inst. Oswaldo Cruz.

[33] Ramalho I.L.C., Araújo FMde C., Cavalcanti L.P.d.G., Braga D.N.M., Perdigão A.C.B., dos Santos F.B., Nogueira F.d.B., da Escóssia K.N.F., Guedes M.I.F. (2018). Dengue 4 in Ceará, Brazil: Characterisation of epidemiological and laboratorial aspects and causes of death during the first epidemic in the state. Mem. Inst. Oswaldo Cruz.

