# Supplementary material for: Retrospective and Contemporary Genomic Surveillance Reveals Genotype-Specific Dengue Lineage Dynamics in Rio de Janeiro
Source: Viruses. 2026 Sep 28;18(10):1070. doi: 10.3390/v18101070 (PMC13709636; doi:10.3390/v18101070)
Supplement: Supplementary file 1 [file viruses-18-01070-s001.zip › viruses-4456745-supplementary.pdf]

Table S1. Epidemiological and sequencing details of DENV samples from Rio de Janeiro, Brazil.

ID	Location	Collection Date	Age	Sex	Sample	CT	Reads	Coverage	Depth of Coverage	Lineages	Acession Number
0069/2013	Niteroi	2013-01-15	17	MASCULINO	serum	29.2	2512958	92.29	33684	4II_B.1.1	EPI_ISL_20485428
0084/2013	Campos dos Goytacazes	2013-01-04	48	MASCULINO	serum	30.52	3045892	92.29	40757.6	4II_B.1.1	EPI_ISL_20485429
0093/2013	Campos dos Goytacazes	2013-01-21	56	FEMININO	serum	24.93	2603745	92.81	35024.7	4II_B.1.1	EPI_ISL_20485430
0451/2021	Barra do Pirai	2021-04-30	46	FEMININO	serum	25.76	2988736	60.04	50450.8	2III_C.1.1	EPI_ISL_20485440
0453/2021	Rio de Janeiro	2021-06-01	20	MASCULINO	serum	22.07	2215208	94.67	27150.4	2III_C.1.1	EPI_ISL_20485441
0454/2021	Barra do Pirai	2021-05-20	6	MASCULINO	serum	22.87	2309090	81.39	28358.7	2III_C.1.1	EPI_ISL_20485442
0459/2021	Rio de Janeiro	2021-06-16	51	MASCULINO	serum	21.44	2207332	88.37	27946	2III_C.1.1	EPI_ISL_20485443
0815/2021	Nova Iguacu	2021-07-19	26	MASCULINO	serum	28.28	2935678	86.86	40375.4	1V_D.2	EPI_ISL_20485436
0819/2021	Rio de Janeiro	2021-07-15	54	MASCULINO	serum	23.48	1900781	73.99	26232.1	2III_C.1.1	EPI_ISL_20485444
0821/2021	Resende	2021-06-30	26	MASCULINO	serum	25.73	2615867	81.37	34427.3	2III_C.1.1	EPI_ISL_20485445
1138/2021	Rio de Janeiro	2021-06-11	19	FEMININO	serum	20.71	2015920	91.1	24907.1	2III_C.1.1	EPI_ISL_20485446
1139/2021	Volta Redonda	2021-06-07	82	MASCULINO	serum	23.74	2077278	85.61	25999.5	2III_C.1.1	EPI_ISL_20485447
1141/2021	Volta Redonda	2021-04-29	54	FEMININO	serum	22.4	2137378	96.21	26289	2III_C.1.1	EPI_ISL_20485448
1142/2021	Rio de Janeiro	2021-04-25	18	MASCULINO	serum	24.51	1767776	86.42	22595.6	2III_C.1.1	EPI_ISL_20485449
1143/2021	Volta Redonda	2021-04-29	39	FEMININO	serum	21.4	2086706	96.42	25761.8	2III_C.1.1	EPI_ISL_20485450
1145/2021	Rio de Janeiro	2021-07-14	11	FEMININO	serum	17.18	2637956	93.57	33479.8	1V_F	EPI_ISL_20485437
1146/2021	Rio de Janeiro	2021-06-16	38	FEMININO	serum	24.79	1948021	78.42	24265.5	2III_C.1.1	EPI_ISL_20485451
1147/2021	Cabo Frio	2021-03-29	28	FEMININO	serum	24.82	2413128	78.67	31997.8	2III_C.1.1	EPI_ISL_20485452
1738/2020	Volta Redonda	2020-04-13	22	MASCULINO	serum	29.5	2364500	66.54	36585.7	2III_C.1.1	EPI_ISL_20485453
1742/2020	Volta Redonda	2020-04-13	45	MASCULINO	serum	23.45	1941110	94.38	24211.1	2III_C.1.1	EPI_ISL_20485454
1758/2020	Parati	2020-04-30	81	MASCULINO	serum	30.84	701415	95.67	8937.8	1V_E.1	EPI_ISL_20485438
2017/2020	Volta Redonda	2020-04-28	9	MASCULINO	serum	22.6	2674928	96.15	33667.8	2III_C.1.1	EPI_ISL_20485455
2032/2020	Volta Redonda	2020-04-29	32	MASCULINO	serum	21.28	2422593	87.23	30845	2III_C.1.1	EPI_ISL_20485456
2035/2020	Volta Redonda	2020-04-29	31	FEMININO	serum	22	3004347	92.61	37317.6	2III_C.1.1	EPI_ISL_20485457
2038/2020	Volta Redonda	2020-04-30	5	FEMININO	serum	25.59	2524692	84.15	33567.9	2III_C.1.1	EPI_ISL_20485458
2066/2020	Parati	2020-04-28		FEMININO	serum	19.16	2630254	94.25	33708.3	1V_E.1	EPI_ISL_20485439
2140/2020	Valenca	2020-05-05	24	MASCULINO	serum	24.49	1826875	77.31	23200.7	2III_C.1.1	EPI_ISL_20485459

2911/2013	Rio das Ostras	2013-04-09	46	MASCULINO	serum	21.66	2028276	92.29	26722.7	4II_B.1.1	EPI_ISL_20485431
2915/2013	Rio de Janeiro	2013-04-10	43	FEMININO	serum	22.96	3254493	92.29	43692.4	4II_B.1.1	EPI_ISL_20485432
2924/2013	Rio de Janeiro	2013-04-10	33	MASCULINO	serum	27.99	2597	71.69	53.29	4II_B.1.1	EPI_ISL_20485427
2930/2013	Seropedica	2013-04-09	20	FEMININO	serum	29.54	1520966	87.82	19768.2	4II_B.1.1	EPI_ISL_20485433
2932/2013	Rio de Janeiro	2013-04-10	22	MASCULINO	serum	27.31	3781	74.84	78.33	4II_B.1.1	EPI_ISL_20485426
2943/2013	Sao Joao de Meriti	2013-04-10	58	MASCULINO	serum	30.36	724352	92.29	9343.11	4II_B.1.1	EPI_ISL_20485434
4396/2018	Rio de Janeiro	2018-04-07	64	FEMININO	serum	20.9	2116075	84.69	26819.7	2II_A.2.1	EPI_ISL_20485435
